# Voltage-gated sodium channels: from roles and mechanisms in the metastatic cell behavior to clinical potential as therapeutic targets

**DOI:** 10.3389/fphar.2023.1206136

**Published:** 2023-06-30

**Authors:** Ana Laura Sanchez-Sandoval, Everardo Hernández-Plata, Juan Carlos Gomora

**Affiliations:** ^1^ Departamento de Neuropatología Molecular, Instituto de Fisiología Celular, Universidad Nacional Autónoma de México, Mexico City, Mexico; ^2^ Medicina Genómica, Hospital General de México “Dr Eduardo Liceaga”, Mexico City, Mexico; ^3^ Consejo Nacional de Humanidades, Ciencias y Tecnologías and Instituto Nacional de Medicina Genómica, Mexico City, Mexico

**Keywords:** sodium channels, therapeutic targets, cancer, metastasis, apoptosis, migration, invasion

## Abstract

During the second half of the last century, the prevalent knowledge recognized the voltage-gated sodium channels (VGSCs) as the proteins responsible for the generation and propagation of action potentials in excitable cells. However, over the last 25 years, new non-canonical roles of VGSCs in cancer hallmarks have been uncovered. Their dysregulated expression and activity have been associated with aggressive features and cancer progression towards metastatic stages, suggesting the potential use of VGSCs as cancer markers and prognostic factors. Recent work has elicited essential information about the signalling pathways modulated by these channels: coupling membrane activity to transcriptional regulation pathways, intracellular and extracellular pH regulation, invadopodia maturation, and proteolytic activity. In a promising scenario, the inhibition of VGSCs with FDA-approved drugs as well as with new synthetic compounds, reduces cancer cell invasion *in vitro* and cancer progression *in vivo*. The purpose of this review is to present an update regarding recent advances and ongoing efforts to have a better understanding of molecular and cellular mechanisms on the involvement of both pore-forming α and auxiliary β subunits of VGSCs in the metastatic processes, with the aim at proposing VGSCs as new oncological markers and targets for anticancer treatments.

## 1 Introduction

### 1.1 Cancer and the metastatic cascade

Cancer is one of the leading causes of morbidity and mortality worldwide, with more than 19 million new cases and near to 10 million cancer-related deaths in 2020, according to the GLOBOCAN 2020 report and, unfortunately, the number of new cases is expected to rise by about 56% over the next 2 decades (Sung et al., 2021).

**FIGURE 1:**
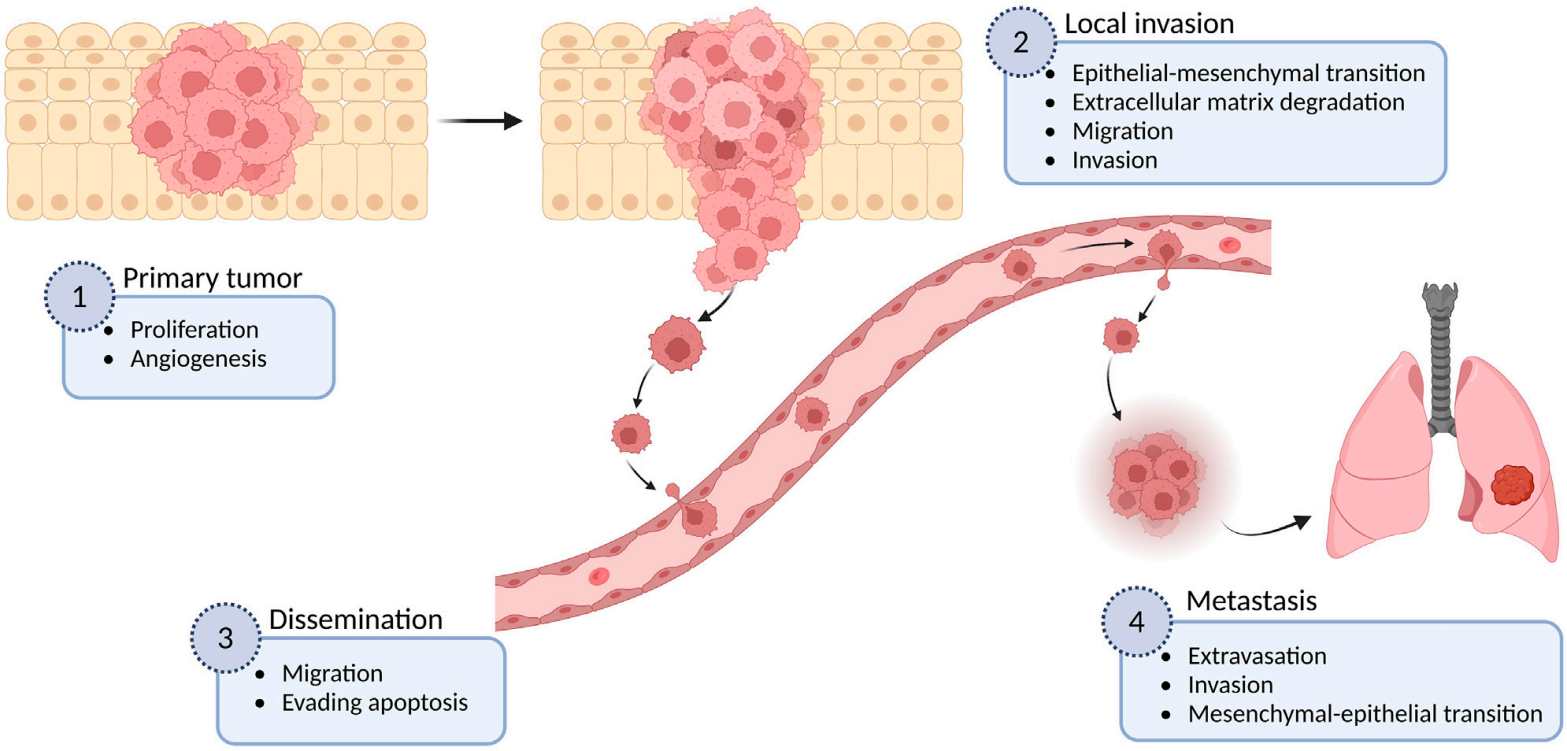
The metastatic cascade. The metastatic process is shown in different stages, as well as some examples of the multiple cellular mechanisms involved in each of these stages. Figure created using BioRender.

Metastasis formation, which defines the ability of cancer cells to spread to distant organs in the body, is responsible for more than 90% of cancer-associated deaths (Spano et al., 2012). The complex process of metastasis is a result of a succession of cell-biology changes, beginning with uncontrolled proliferation and local invasion of surrounding normal tissue, then intravasation of cancer cells into vessels (blood and lymphatic), transit and survival through the lymphatic or hematogenous systems, arrest in capillary beds of distant tissues, followed by extravasation, attachment and proliferation of cancer cells at secondary organs (micrometastasis), and finally the growth of new colonies into macroscopic tumors (Hanahan and Weinberg, 2011) (Figure 1). This multistep process, often called the invasion-metastasis cascade, involves the dysregulation of numerous proteins including several members of the ion channel superfamily (Talmadge and Fidler, 2010; Litan and Langhans, 2015). In particular, voltage-gated sodium channels (VGSCs or Na_V_ channels) are functionally expressed in many types of cancer, comprising astrocytoma, breast, cervix, colon, gastric, leukemia, lung, melanoma, mesothelioma, neuroblastoma, ovary, prostate, and thyroid cancer (Blandino et al., 1995; Allen et al., 1997; Gu et al., 1997; Laniado et al., 1997; Bordey and Sontheimer, 1998; Smith et al., 1998; Diss et al., 2001; Roger et al., 2003; Fraser et al., 2004; Fraser et al., 2005; Onganer and Djamgoz, 2005; Ou et al., 2005; Fulgenzi et al., 2006; Diaz et al., 2007; Roger et al., 2007; Carrithers et al., 2009; Gao et al., 2010; House et al., 2010; Hernandez-Plata et al., 2012; Campbell et al., 2013; Xing et al., 2014; Huang et al., 2015; Xia et al., 2016; Li et al., 2022), where their upregulation has been correlated with the potentiation of cellular migration and invasiveness, as well as with other metastatic cell behaviors (MCBs) including proliferation, invadopodia formation, endocytosis, angiogenesis, and resisting apoptosis (Mycielska et al., 2003; Krasowska et al., 2004; Onganer and Djamgoz, 2005; Carrithers et al., 2009; Andrikopoulos et al., 2011; Xing et al., 2014).

### 1.2 Voltage-gated sodium channels: structure and function

**FIGURE 2:**
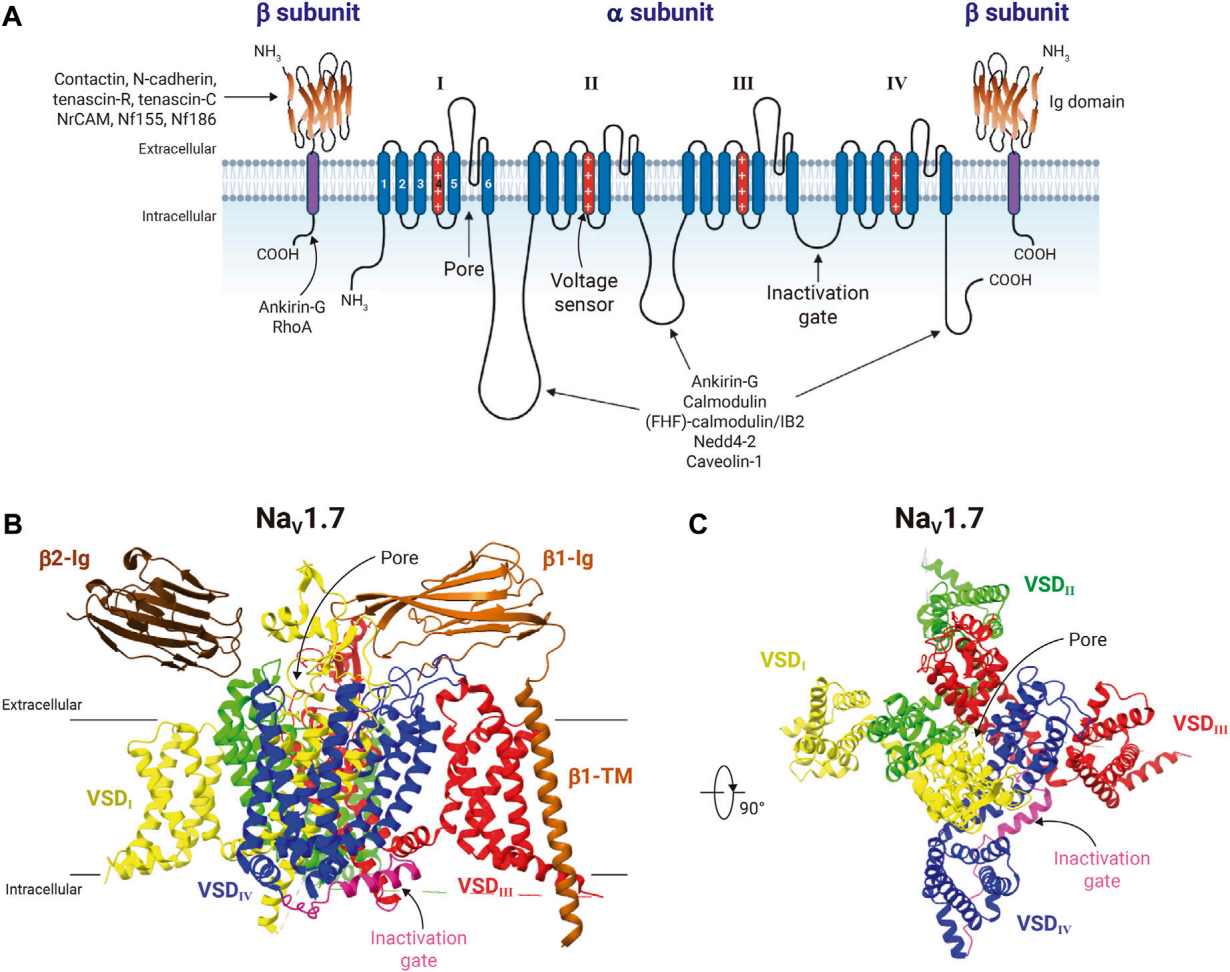
Structure of voltage-gated sodium-channels. **(A)** General schematic representation of the voltage-gated sodium-channel protein complex. The VGSC α-subunit is illustrated together with two β-subunits. The α-subunit is formed by four homologous domains (from I to IV), each with a voltage sensor domain (VSD) composed by transmembrane (TM) segments 1 to 4. The transmembrane segments 4 of each domain (shown in red), characterized by containing amino acids with positive charge every 3 positions, function as the voltage sensors of the channel; and segments 5 and 6 are the porelining. The intracellular loop between domains III and IV contains the inactivation gate motif Ile/Phe/Met (IFM). The β-subunits consist of a single TM segment and an immunoglobulin-like fold (Ig) extracellular domain, which interacts with the extracellular loops of the α-subunit. Different proteins that interact with α- and β-subunits of VGSC are also shown. Figure created using BioRender. **(B)** Cryo-electron microscopy structure of human Na_V_1.7 in complex with β1 and β2 in a side view (PDB: 6J8J). Na_V_1.7 is colored by repeats as indicated. The inactivation gate (with the IFM motif) is shown in pink. Transmembrane and Ig domains of β1 are shown in ocher color, whereas for β2 only the extracellular Ig domain was resolved (brown). **(C)** Extracellular view of Na_V_1.7 α-subunit. β-subunits were removed for clarity purposes. Structure figures were prepared in ChimeraX.

The family of VGSCs consists of transmembrane protein complexes conformed by a pore-forming functional α-subunit (∼230 kDa) typically associated with one or more non-pore forming β-subunits (∼25–45 kDa) (Isom, 2001; Catterall et al., 2005); and they are responsible for the action potential initiation and propagation in excitable cells, including nerves, muscles, and neuroendocrine cells (Hille, 2001). The VGSCs α-subunits are formed by approximately 2000 amino acid residues organized in four homologous domains, each one comprising six transmembrane segments (S1-S6) (Figure 2A). So far, nine mammalian VGSCs α-subunits, Na_V_1.1 to Na_V_1.9, encoded by the genes *SCN1A-SCN5A* and *SCN8A-SCN11A*, have been identified, cloned, and characterized (Goldin et al., 2000; Alexander et al., 2021) (Table 1). The *SCN7A* and *SCN6A* genes code for the 10th sodium channel protein (named Na_X_) that is not voltage-gated and is involved in sodium-level sensing (Noda and Hiyama, 2015; Noland et al., 2022). During the last 12 years the use of X-ray crystallography, but mainly the cryo-electron microscopy techniques have provided amazing atomic resolution about the structural architecture, activation, inactivation, voltage-sensing, and pharmacology of VGSCs (Payandeh et al., 2011; Pan et al., 2018; Jiang et al., 2020; Jiang et al., 2022; Fan et al., 2023), (Figure 2B). These different sodium channels have similar structural and functional properties, but their expression is cell type-specific, and they have distinct regulatory and pharmacological characteristics (Goldin, 2001; Catterall et al., 2005; Catterall, 2012; Shen et al., 2019; Catterall et al., 2020). According to their sensitivity to the neurotoxin tetrodotoxin (TTX), the VGSCs can be classified into two main groups: the TTX-sensitive channels, including Na_V_1.1-Na_V_1.4, Na_V_1.6, and Na_V_1.7, which are inhibited by low nanomolar concentrations of TTX; and the TTX-resistant channels Na_V_1.5, Na_V_1.8, and Na_V_1.9, that are inhibited by TTX at micromolar concentrations (Table 1).

**TABLE 1:** Human voltage-gated sodium channel α-subunits.

Type	Gene symbol	% Identity	Primary tissue	TTX-sensitivity (IC_50_)^a^
Na_V_1.1	*SCN1A*	----	CNS neurons	4.1 nM
Na_V_1.2	*SCN2A*	87.90	CNS neurons	14 nM
Na_V_1.3	*SCN3A*	84.74	CNS neurons	5.3 nM
Na_V_1.6	*SCN8A*	77.62	CNS neurons	2.3 nM
Na_V_1.7	*SCN9A*	77.50	PNS neurons	36 nM
Na_V_1.4	*SCN4A*	71.17	SkM	7.6 nM
Na_V_1.5	*SCN5A*	64.95	Heart	1 µM
Na_V_1.8	*SCN10A*	58.55	DRG neurons	73.3 µM^b^
Na_V_1.9	*SCN11A*	52.65	DRG neurons	>30 µM^c^

Percent identity (shown *versus* Na_V_1.1) was calculated by using Clustal Omega 12.1 Multiple Sequence Alignment tool for the full length of the following PDBs: Na_V_1.1, P35498; Na_V_1.2, Q99250; Na_V_1.3, Q9NY46; Na_V_1.4, P35499; Na_V_1.5*,* Q14524; Na_V_1.6*,* Q9UQD0; Na_V_1.7*,* Q15858; Na_V_1.8*,* Q9Y5Y9; and Na_V_1.9*,* Q9UI33. CNS and PNS, central and peripheral nervous system; SkM, skeletal muscle; DRG, Dorsal Root Ganglion.

^a^ Tsukamoto et al., 2017.

^b^ Akiba et al., 2003.

^c^ Vanoye et al., 2013.

Sodium current through VGSCs displays two main components: the characteristic “transient” component (*I*_Na_T), responsible for the fast sodium influx into the cells during action potentials, and the “persistent” component (*I*_Na_P), which is a small fraction of the corresponding *I*_Na_T (∼10%) that shows little or no inactivation at all (Crill, 1996; Djamgoz and Onkal, 2013). The persistent current has a crucial relevance in functionality of neurons with repetitive firing activity, where low but constant sodium influx maintains the membrane potential subtly depolarized facilitating the action potential generation (Osorio et al., 2010; Nakamura and Jang, 2022).

Among the great diversity of proteins related to VGSCs, it has been shown that the C-terminal region of several VGSCs channels interacts with the fibroblast growth factor homologous factor (FHF)-calmodulin, regulating current density, availability, frequency -dependent inhibition, and resurgent currents (Goldfarb, 2005; Pitt and Lee, 2016; Mahling et al., 2021). In addition, the clustering of VGSCs in the membrane is functionally relevant (Nirenberg and Yifrach, 2019); this property can be regulated by interactions with the ubiquitin ligases nedd4 and nedd4-2 (van Bemmelen et al., 2004), and other cytoskeletal proteins such as ankyrin, neurofascin, and spectrin (Liu et al., 2020). Evidence from central nervous system research have shown that Na_V_1.2 channels interact with the anchoring protein AKAP, which in turn recruits the kinases PKA and PKC. Serine residues phosphorylation made by PKA in the intracellular loop joining DI and DII domains of the α-subunit reduces the sodium currents transported by VGSCs, and the PKC activity increases this effect (Cantrell et al., 2002).

Similarly, tyrosine phosphorylation by the Src family kinase Fyn produces a depolarizing shift of the steady-state inactivation on Na_V_1.5 channels. Single amino acid substitution assays have revealed the importance of the Y1495 located in the intracellular linker of domains III and IV for this effect (Ahern et al., 2005). On Na_V_1.2 channel, the Fyn kinase accelerates the inactivation which reduces the sodium current and shifts the voltage dependence of the inactivation to more negative potentials (Ahn et al., 2007).

On the other hand, β subunits of VGSCs are composed by a prominent N-terminal extracellular immunoglobulin-like fold related to the L1 family of cell adhesion molecules, a single transmembrane segment, and a short intracellular carboxyl-terminal region. These proteins modulate the voltage sensitivity, gating kinetics, and trafficking of VGSCs, but they also play functional roles independently of α subunits in a variety of cellular processes in both excitable and non-excitable cells, including cancer cells (Brackenbury and Isom, 2011). Four genes (*SCNB1-SCNB4*) encode for five VGSCs β-subunits: β1a and β1b (generated by alternative splicing), β2, β3, and β4 (Table 2). β1 and β3 resemble each other in amino acid sequence and are associated non-covalently with α subunits. In contrast, β2 and β4 form disulfide bonds with α subunits and have similar amino acid sequences (Isom et al., 1992; Isom et al., 1995; Morgan et al., 2000; Yu et al., 2003). Additionally, the VGSCs β-subunits are able to interact with intracellular molecules (e.g., ankyrin_G_ and ankyrin_B_), and also act as cell adhesion molecules (CAMs) through their immunoglobulin extracellular domain, interacting with each other (homophilic interaction) or other CAMs (heterophilic interaction, e.g., contactin, NF-155 and NrCAMs), either expressed in the same cell (*cis*-interaction) or with CAMs from other cells (*trans*-interaction), as well as with components of the extracellular matrix (e.g., tenascin-C and tenascin-R; Figure 2A) (Brackenbury and Isom, 2011). In addition, abnormalities in expression of VGSCs β-subunits, such as mutations or gene expression dysregulations are implicated in several inherited pathologies, including some epilepsies and heart arrhythmias and syndromes (O'Malley and Isom, 2015; Edokobi and Isom, 2018).

**TABLE 2:** Human voltage-gated sodium channel β-subunits.

Type	Gene symbol	% Identity	Main functions^a^ ^,^ ^b^	Disease involvement^c^ ^,^ ^d^
Na_V_β1	*SCN1B*	----	Enhances the presence of the α subunit at the cell surface. Modulates channel gating. Promotes cell adhesion.	Generalized epilepsy with febrile seizures. Developmental and epileptic encephalopathy. Dravet Syndrome. Brugada Syndrome. Atrial fibrillation. Sudden death.
Na_V_β1b	*SCN1B*	75.47	Secreted cell adhesion molecule involved in neuronal migration and pathfinding during brain development. Stimulates neurite outgrowth.	Atrial fibrillation. Long QT syndrome. Idiopathic epilepsy.
Na_V_β3	*SCN3B*	49.05	Modulates channel gating kinetics. Causes unique persistent sodium currents. Promotes cell adhesion.	Brugada syndrome. Atrial fibrillation. Sudden infant death syndrome.
Na_V_β2	*SCN2B*	20.00	Promotes trafficking of α subunit to the plasma membrane. Modulates channel gating. Intracellular domain acts as a transcriptional activator for Na_V_1.1. Promotes cell adhesion.	Brugada syndrome. Atrial fibrillation. Idiopathic small fiber neuropathy.
Na_V_β4	*SCN4B*	16.67	Modulates channel gating kinetics. Crucial for the generation of “resurgent currents”. Promotes cell adhesion.	Long QT syndrome. Atrial fibrillation.

Percent identity (shown *versus* Na_V_β1) was calculated by using Clustal Omega 12.1 Multiple Sequence Alignment tool for the full length of the following PDBs: Na_V_β1, Q07699; Na_V_β1b, Q07699-2; Na_V_β2, O60939; Na_V_β3*,* Q9NY72; Na_V_β4*, Q8IWT1.*

^a^ Bouza and Isom, 2018.

^b^ Patino and Isom, 2010.

^c^ O'Malley and Isom, 2015.

^d^ Salvage, et al., 2023.

### 1.3 VGSCs expression in cancer

Over the last 25 years, numerous studies have documented the *novo* expression/overexpression of multiple VGSC subunits in several types of cancer and their contribution to different malignant behaviors, remarkably migration and invasion. In addition, the predominantly upregulated α subunit appears to be cancer-type specific (Table 3), and some reports have evidenced the preferential expression of variants generated by alternative splicing of the corresponding coding mRNAs. Importantly, neonatal splice variants of VGSCs are abundantly expressed in breast cancer (BCa), cervical cancer, colon cancer, neuroblastoma, non-small-cell lung cancer, and prostate cancer (PCa) (Diss et al., 2001; Fraser et al., 2005; Ou et al., 2005; Roger et al., 2007; Guzel et al., 2018; Lopez-Charcas et al., 2018). This is consistent with the large amount of re-expression of embryonic genes during oncogenesis (Monk and Holding, 2001).

**TABLE 3:** VGSCs α-subunit expression and role in different cancer cells.

Expressed VGSCs α subunit	Cancer type	Metastatic characteristic involved	References
Na_v_1.1	Glioma	ND	Schrey et al. (2002)
	Ovary	ND	Gao et al. (2010)
Na_v_1.2	Cervix	ND	Diaz et al. (2007)
	Glioma	ND	Schrey et al. (2002)
	Mesothelioma	Migration	Fulgenzi et al. (2006)
	Neuroblastoma	ND	Vetter et al. (2012)
	Non-small cell lung	Invasion	Roger et al. (2007)
	Ovary	ND	Gao et al. (2010)
	Prostate	ND	Diss et al. (2001)
Na_v_1.3	Glioma	ND	Schrey et al. (2002)
	Leukemia	Invasion	Huang et al. (2015)
	Neuroblastoma	ND	Vetter et al. (2012)
	Non-small cell lung	Invasion	Roger et al. (2007)
	Ovary	ND	Gao et al. (2010)
	Prostate	ND	Diss et al. (2001)
Na_v_1.4	Cervix	ND	Diaz et al. (2007)
	Neuroblastoma	ND	Vetter et al. (2012)
	Ovary	ND	Gao et al. (2010)
Na_v_1.5	Astrocytoma	Migration, invasion, proliferation, evading apoptosis	Xing et al. (2014)
	Breast	Migration	Fraser et al. (2005), Brackenbury et al. (2007)
		Invasion	Fraser et al. (2005), Brackenbury et al. (2007), Gao et al. (2009), Gillet et al. (2009), Brisson et al. (2011), Brisson et al. (2013)
		Invadopodia formation	Brisson et al. (2013)
		Galvanotaxis	Fraser et al. (2005)
		Endocytosis	Fraser et al. (2005)
	Colon	Invasion	House et al. (2010), Guzel et al. (2018), Lopez-Charcas et al. (2022)
	Leukemia	Invasion	Fraser et al. (2004)
	Neuroblastoma	ND	Ou et al. (2005), Vetter et al. (2012)
	Non-small cell Lung	Invasion	Roger et al. (2007)
	Ovary	Migration, invasion	Gao et al. (2010)
Na_v_1.6	Cervix	Invasion	Hernandez-Plata et al. (2012), Lopez-Charcas et al. (2018)
	Glioma	ND	Schrey et al. (2002)
	Leukemia	Invasion	Fraser et al. (2004), Huang et al. (2015)
	Melanoma	Podosome formation, invasion	Carrithers et al. (2009)
	Mesothelioma	Migration	Fulgenzi et al. (2006)
	Non-small cell Lung	Invasion	Roger et al. (2007)
	Osteosarcoma	Evading apoptosis	Hernandez-Muñoz et al. (2016)
	Prostate	Invasion	Nakajima et al. (2009)
	Thyroid	Invasion, proliferation	Li et al. (2022)
Na_v_1.7	Cervix	Invasion	Hernandez-Plata et al. (2012)
	Gastric	Invasion	Xia et al. (2016)
	Endometrial	Invasion, proliferation	Liu et al. (2019)
	Leukemia	Invasion	Fraser et al. (2004), Huang et al. (2015)
	Mesothelioma	Migration	Fulgenzi et al. (2006)
	Neuroblastoma	ND	Vetter et al. (2012)
	Non-small cell lung	Invasion	Roger et al. (2007), Campbell et al. (2013)
	Ovary	ND	Gao et al. (2010)
	Prostate	Invasion	Nakajima et al. (2009)
Na_v_1.8	Non-small cell Lung	Invasion	Roger et al. (2007)
	Ovary	ND	Gao et al. (2010)
Na_v_1.9	Leukemia	ND	Fraser et al. (2004)

ND, not determined.

The neonatal Na_V_1.5 isoform has been characterized in BCa and colon cancer cells and found to have distinct electrophysiological properties compared to the adult variant; e.g., it is more active and allows a higher influx of sodium ions into the cell (Onkal et al., 2008; Guzel et al., 2018). The neonatal Na_V_1.5 variant differs from the adult isoform by 7 amino acids in the S3 and the S3/S4 linker from domain I, and is also found in neuroblastoma (Ou et al., 2005), where another splice variant of Na_V_1.5 lacking the full exon 18 (Na_V_1.5-Δ18) has been shown to generate a functional sodium channel, even though this exon encodes 54 amino acids in the intracellular loop between domains II and III (Ou et al., 2005). On the other hand, in cervical cancer, leukemia, and melanoma, the expression of the Na_V_1.6-Δ18 splice variant is promoted (in this case, exon 18 encodes a 41-amino acid portion of the S3 and S4 segments of domain III). This isoform has been reported to have an intracellular localization, rather than being expressed in the plasma membrane, and it appears to contribute to the invadopodia formation and the invasive potential of these cells by a different mechanism than the sole influx of sodium through the plasma membrane (Carrithers et al., 2009; Lopez-Charcas et al., 2018).

Nowadays it is well known that cancer cells generally possess a more depolarized membrane potential with respect to normal healthy tissue, which has been correlated with cancer progression, as the depolarized membrane potential itself plays important roles in cell cycle progression, DNA synthesis, mitosis, proliferation, migration, and differentiation (Lobikin et al., 2012; Yang and Brackenbury, 2013). In this context, several studies have demonstrated that intracellular sodium concentration is higher in cancer cells compared with normal cells, and such increased concentration is related to mitogenesis and oncogenesis (Cameron et al., 1980; Ouwerkerk et al., 2007). Moreover, it has been proposed that the *I*_Na_P is the main responsible for the elevation of the intracellular sodium concentration and the consequent potentiation of several MCBs, as *I*_Na_P significantly manifests itself under a variety of physiological and pathophysiological conditions, including cancer (Djamgoz and Onkal, 2013). In addition, almost all the reported studies have revealed the role of VGSCs in metastatic behaviors of cancer cells by means of the modulation of their activity (Roger et al., 2003; Fraser et al., 2005; Brackenbury and Djamgoz, 2006; Gillet et al., 2009), employing pharmacological drugs or natural toxins to modulate the channel opening or closing, and the corresponding sodium ion flow**.** More recently, the regulation of gene expression using small interference RNAs (siRNAs) has allowed to modulate the amount of functionally expressed channels and characterize their role more precisely (Brisson et al., 2011; Driffort et al., 2014). The role of VGSC β-subunits have also been studied, although less extensively, in breast, cervical, prostate, and thyroid cancer (Diss et al., 2008; Chioni et al., 2009; Jansson et al., 2012; Jansson et al., 2014; Nelson et al., 2014; Bon et al., 2016; Gong et al., 2018; Sanchez-Sandoval and Gomora, 2019; Haworth et al., 2022), in which they have an important role in several MCBs as well (Table 4).

**TABLE 4:** VGSCs β-subunit expression and role in different cancer cells.

Expressed VGSCs β subunit	Cancer type	Metastatic characteristic involved	References
NaVβ1	Breast	Migration, adhesion	Chioni et al. (2009)
		Invasion, process length, angiogenesis, evading apoptosis	Nelson et al. (2014)
		Invasion	Bon et al. (2016)
	Cervix	Proliferation, migration	Sanchez-Sandoval and Gomora (2019)
	Non-small cell lung	ND	Roger et al. (2007)
	Prostate	ND	Diss et al. (2008)
NaVβ2	Breast	ND	Chioni et al. (2009), Bon et al. (2016)
	Cervix	ND	Sanchez-Sandoval and Gomora (2019)
	Neuroblastoma	ND	Vetter et al. (2012)
	Prostate	Migration, invasion, process length, proliferation	Jansson et al. (2012), Jansson et al. (2014)
NaVβ3	Cervix	Proliferation, apoptosis	Sanchez-Sandoval and Gomora (2019)
	Hepatic	Evading apoptosis	Li et al. (2020)
	Neuroblastoma	ND	Vetter et al. (2012)
	Non-small cell lung	ND	Roger et al. (2007)
	Prostate	ND	Diss et al. (2008)
NaVβ4	Breast	Migration, invasion, podosome formation	Bon et al. (2016)
	Cervix	Invasion	Sanchez-Sandoval and Gomora (2019)
	Thyroid	ND	Gong et al. (2018)
	Prostate	ND	Diss et al. (2008)

ND, not determined.

Despite all the new evidence of the VGSCs overexpression and functional contribution to numerous MCBs in different types of cancer, the entire mechanisms by which the sodium channel upregulation enhances this phenotype has not been completely elucidated (Lopez-Charcas et al., 2021). However, the evidence compiled hitherto provide an approach to understand the pathobiology of cancer and could be useful in a near future to fight the metastatic phenotype of cancer.

The aim of the present review is to collect the newest information that conforms to the state of the art in this field of knowledge, focusing on the role of VGSCs in different metastatic cell behaviors including migration, invasion, proliferation, angiogenesis, endocytosis, and evasion of apoptosis, as well as the latest revealed molecular mechanisms of action. Most evidence included in this review come from studies in cancer, nevertheless some non-cancer examples that could explain phenomena observed in cancer cells are also reviewed.

## 2 Metastatic cell behaviors: role of VGSCs

The complex metastatic process involves the transformation of cells through multiple steps in which a large number of cellular and molecular mechanisms are involved. There is a great variety of proteins orchestrating this complex process, among which are the VGSCs, whose participation in each of the characteristics of the metastatic process is detailed below.

### 2.1 Proliferation

One of the most fundamental characteristics of cancer cells is their ability to maintain uncontrolled proliferation, which is carefully controlled in normal cells by different signals, including growth factors and hormones, in order to ensure the homeostasis of tissue architecture and function (Hanahan and Weinberg, 2011). While several studies have reported no effect on cell proliferation by VGSCs (e.g., Fraser et al., 1999; Fulgenzi et al., 2006; Roger et al., 2007; Gao et al., 2010; Hernandez-Plata et al., 2012; Huang et al., 2015), a few others have shown the opposite, demonstrating once more the tissue-specific functionality of the channels, as well as the variability given by the study model.

By inhibiting the sodium currents with VGSCs-blockers, including phenytoin analogues and riluzole, the proliferation of PCa cell lines decreases without affecting cell viability (Anderson et al., 2003; Driffort et al., 2014; Rizaner et al., 2020). Also, cell proliferation is inhibited in U251 astrocytoma cells when Na_V_1.5 expression is downregulated by siRNAs (Xing et al., 2014). In the same sense, *in vitro* proliferation of gastric cancer cell lines is markedly slower when they are incubated in the presence of TTX, EIPA (5-(N-ethyl-N-isopropyl) amiloride; a Na^+^/H^+^ exchanger 1 inhibitor), or when they are transfected with siRNAs against Na_V_1.7, consistent with a reduced xenograft tumor growth of stable Na_V_1.7 knockdown BGC-823 cells compared to scramble-transfected control cells (Xia et al., 2016), and smaller tumor size generated by MDA-MB-231 cells in phenytoin-treated animals (Nelson et al., 2015). Very recently, it has been shown that knocking down Na_V_1.6 expression significantly inhibited the proliferation, epithelial–mesenchymal transition, and invasion of follicular thyroid carcinoma (FTC), through the JAK/STAT signaling pathway (Li et al., 2022), which is associated with crucial pathological processes of tumors including cell proliferation, invasion, and apoptosis (Hu et al., 2021). Remarkably, in this work the migration behavior of FTC cells was not explored and the proposed Na_V_1.6-activated signaling pathway seems to be the same for the proliferation and invasiveness of FTC. Since tumor cell proliferation mechanisms can be different *in vitro versus in vivo*, the effect of different drugs, toxins, and gene expression regulation on Na_V_ channels in this cell function should be considered carefully, as several cross-effects involving other proteins may be present.

Proliferation of non-cancer cells has also been demonstrated to be regulated by VGSCs activity. Vascular endothelial growth factor-induced proliferation in human umbilical vein endothelial cells (HUVECs) is reduced when VGSCs activity is blocked by TTX (Andrikopoulos et al., 2011). Similarly, proliferation of embryonic cardiomyocytes in zebrafish is dependent of the cardiac sodium channel ortholog scn5Lab (Bennett et al., 2013).

The underlying molecular mechanism linking sodium channels to cell proliferation is not completely clear yet, however it appears to be related to a higher response to growth factors and/or hormones. A strong relationship between several of these molecules and the VGSCs activity has been reported, including the epidermal growth factor (EGF) (Liu et al., 2007; Uysal-Onganer and Djamgoz, 2007; Ding et al., 2008; Campbell et al., 2013), the vascular endothelial growth factor (VEGF) (Andrikopoulos et al., 2011; Pan et al., 2012; Sun and Ma, 2013), the insulin-like growth factor (IGF) (Yanagita et al., 2011), the nerve growth factor (NGF) (Brackenbury and Djamgoz, 2007), β-estradiol (Fraser et al., 2010; Hu et al., 2012), and dihydrotestosterone (Diss et al., 2008).

Additionally, the role of VGSCs β-subunits in the proliferation of different cancer cells has also been studied. Overexpressing β2 in stably transfected LNCaP cells *in vitro* has no effect on the proliferation rate in the first days, however, it suppresses the contact inhibition of proliferation, allowing the cells to keep proliferating even in saturation density (Jansson et al., 2012; Jansson et al., 2014). Likewise, overexpression of β1 led to an increased proliferation of SiHa cervical cancer cells, but no effects are observed in HeLa and CaSki cells, evidencing the importance of specific cellular characteristics of one type of cells and not a general behavior in cervical cancer (Sanchez-Sandoval and Gomora, 2019). In BCa, the role of β-subunits is even more controversial. For instance, the proliferation of MDA-MB-231 cells overexpressing β1 is reduced *in vitro* compared to control cells (Chioni et al., 2009), on the other hand, the tumors produced by these cells in an orthotopic model *in vivo* are bigger than those produced by control cells, suggesting a promoted proliferation exerted by β1 (Nelson et al., 2014).

### 2.2 Angiogenesis

Tumor cells, like normal tissue, require oxygen and nutrients as well as the ability to evacuate metabolic wastes. Given their uncontrolled proliferation, malignant tumoral cells address these needs throughout the activation of angiogenesis, promoting tumor-associated neovasculature, which is induced very early during the development of invasive cancers. These new vessels are typically anomalous, with an excessive vessel branching, leakiness, distorted, and enlarged structures and irregular blood flow (Hanahan and Weinberg, 2011). The formation of new vessels is triggered by factors produced and secreted by tumoral cells; most types of human cancers express elevated levels of VEGF, the major mediator of tumor angiogenesis (Kerbel, 2008), whose expression is induced by several environmental and molecular factors, including hypoxia and VGSCs’ activity, among others. For example, it has been proved that Na_V_1.5 channels potentiate the angiogenesis process induced by VEGF through the activation of the PKCα-B-RAF-ERK1/2 signaling pathway, in HUVECs; the tubular differentiation on Matrigel (a commercial basement membrane matrix), is significantly inhibited by the exposure of these cells to TTX (Andrikopoulos et al., 2011).

On the other hand, in epithelial BCa cells, the induced expression of modulatory β1 subunit promotes the VEGF secretion and increases the formation of new vascular vessels, suggesting a contributing role of β1 proteins through the modulation of cellular adhesion as CAMs (Nelson et al., 2014).

### 2.3 Epithelial-mesenchymal transition

About 90% of cancers originate from epithelial tissues, thus designed as carcinomas. In order to invade surrounding tissues, primary tumor cells must undergo dedifferentiation or transdifferentiation processes. The epithelial-to-mesenchymal transition (EMT) takes place through gradual changes in which multiple tumoral cell subpopulations proliferate to increase the tumor size. From an epithelial to a complete mesenchymal phenotype, several steps modulated by different specific cellular niches must occur (Pastushenko et al., 2018). This phenomenon, which is normal in embryonic morphogenesis and wound healing, can be activated transiently, stably, or gradually by carcinoma cells. The EMT process is characterized by loss of cell-cell and cell-matrix adherent junctions and it is typically associated with the transformation from a polygonal apical-basal polarized morphology to a more spindle-like shape capable of degrading and invading the extracellular matrix of surrounding tissues firstly, and secondary distant sites latter (Davis et al., 2014; Ye and Weinberg, 2015). At the molecular level, it has been shown that EMT is under the control of transcription factors such as SNAIL, bHLH and ZEB factors (Lamouille et al., 2014). In this regard, it has been shown that the activity of Na_V_1.5 channels can induce the expression of SNAI1 and ZEB1 transcription factors in breast cancer cells; and the downregulation of this channel reverts the mesenchymal phenotype (Gradek et al., 2019).

The EMT involves the downregulation of characteristic epithelial proteins such as E-cadherin, cytokeratin, and laminin-1, while increasing the expression of mesenchymal phenotype molecules, including N-cadherin, vimentin, and fibronectin (Christofori, 2006), which in turn leads to a diminished affinity for epithelial tissue and higher affinity for mesenchymal cells. As a result, cell detachment, migration and invasion are facilitated (Yilmaz and Christofori, 2009). Moreover, as mentioned before, the EMT process can be progressive, as some cancer cells conforming the tumoral mass in transition can express protein markers from both epithelial and mesenchymal phenotypes; this plasticity in protein expression is directly related to the response to pharmacological treatments, and development of drug resistance (Navas et al., 2020).

Generated changes from the benign phenotype can progress to an invasive cancer and then dissemination of malignant cells to distant organs resolving the selection pressure imposed by the tumor environment, leading to the generation of metastasis (Reeves et al., 2018). In these processes, several VGSCs (both α and β subunits) have been shown to play important roles, which are described below.

#### 2.3.1 Adhesion/detachment

Adhesion and detachment of cells to the extracellular matrix and the neighboring cells is a critical step in the EMT; these processes are mediated by the coordinated expression of numerous proteins. In 2008, Palmer and colleagues demonstrated the participation of VGSCs in cell adhesion of highly metastatic prostate cancer cells. Using a single cell adhesion measuring apparatus (SCAMA), which measures the negative pressure needed to detach a cell from the substrate, they found that when blocking the activity of VGSCs with TTX, the strength of cell adhesion of the highly metastatic PCa cells, Mat-LyLu, and PC-3M, was significantly increased, whereas no difference was detected with the weakly metastatic AT-2 cells, and the normal human prostatic epithelial cells PNT2-C2, that do not express functional VGSCs (Palmer et al., 2008). TTX treatment also increases cell adhesion in highly metastatic MDA-MB-231 breast cancer cells and in HUVECs, both expressing functional VGSCs (Pan and Djamgoz, 2008; Andrikopoulos et al., 2011). However, the precise mechanism by which the channel activity modulates cell adhesion has not been totally clarified so far.

In this regard, β-subunits of VGSCs can also participate in cellular adhesion functioning as CAMs through their immunoglobulin extracellular domain, with each other or with other CAMs. β1 and β2 interact in a trans-homophilic way in transfected *Drosophila* S2 cells, resulting in cellular aggregation; in addition, the β-subunits participating in cell to cell contact points promote the recruitment of ankyrin intracellularly (Malhotra et al., 2000), forming structural protein complexes implying interactions with the cytoskeleton and signaling pathways, as occurs in the development of the central nervous system (Brackenbury et al., 2008). In the same tissue, β1 subunit interacts not only with β2, but also with contactin, N-cadherin, tenascin-R, NrCAM, Nf155, and Nf186, facilitating the recruitment and concentration of VGSCs at axon initial segments and nodes of Ranvier (Xiao et al., 1999; Malhotra et al., 2000; Ratcliffe et al., 2001; Malhotra et al., 2004; McEwen and Isom, 2004). Also, β2 interacts with tenascin-R and tenascin-C (Srinivasan et al., 1998), however, it does not interconnect with contactin (McEwen and Isom, 2004). Interestingly, β3 interacts with Nf186 (Ratcliffe et al., 2001), but it does not mediate homophilic binding in transfected *Drosophila* S2 cells, while it does when expressed in mammalian HEK-293 cells, probably due to differences in glycosylation levels, as these has been shown to be crucial for ensuring the correct orientation of the Ig domain (McEwen and Isom, 2004; Yereddi et al., 2013).

In BCa, the weakly metastatic MCF-7 cells express higher levels of β1, β2, and β4 subunits and are more adhesive to the substrate than highly metastatic MDA-MB-231 cells, supporting the role of these proteins as CAMs. Downregulation of β1 with siRNAs in MCF-7 cells produces a significant reduction in single-cell adhesion. Furthermore, overexpression of β1 in MDA-MB-231 cells increased cell-cell adhesion, most likely due to augmented trans-homophilic interactions (Palmer et al., 2008; Chioni et al., 2009). Contrasting results have been reported for the β2 subunit in prostate cancer, where stably overexpression of β2 in LNCaP cells increased migration and invasiveness, while selectively enhances the adhesion to vitronectin, laminin, and Matrigel, also improving the ability of the cells to remain attached to rat embryonic dorsal root ganglion F11 cells (Jansson et al., 2012; Jansson et al., 2014).

The selective interactions with specific molecules of the extracellular matrix and other cells might promote migration and/or invasion during cancer progression. CAMs play distinct roles in context-dependent cell-cell and cell-extracellular matrix adhesion, including the ability to transduce the signals from the environment and trigger intracellular responses. Conformational changes in CAMs can remodel nascent or focal adhesions and generate tension, whereas coordinated assembly and disassembly of these adherent structures generate forces of cellular movement (Janiszewska et al., 2020).

The β4 subunit also participates in cell-cell interactions, as a CAM. The β4 N-terminal extracellular domains of adjacent cells interact with each other parallelly stablishing disulfide bonds between cysteine residues, and hydrophobic and hydrogen-bonding interactions, which form β4-cis trans homophilic dimers that modulate cellular adhesion, multicellular aggregation, and increase the number of F-actin filopodia-like protrusions in the membranal reorganization. Typically, the overexpression of β4 subunit into a heterologous system increases cell adhesion (Miyazaki et al., 2007; Shimizu et al., 2017).

#### 2.3.2 Morphology and migration

The migratory and invasive phenotype of cancer cells are the key parameters of the metastatic cascade, as the cells must travel from primary tumor to distant organs. This movement can respond to chemical factors (chemotaxis) or physical factors and varies in type and quantity from cancer to cancer. In all cases, dramatic reorganization of the cytoskeleton is necessary to produce the morphological changes needed in the cell to promote mobilization. Mesenchymal migration is characterized by the formation of a fibroblastoid spindle-shape morphology, increasing the cell-matrix interactions and promoting the release from the primary tumor, as described before. Furthermore, amoeboid migration involves a less adhesive cell-matrix interactions and more diffuse structure of the cytoskeleton, usually mediated by RhoA activity and active myosin-actin contractions, allowing the cells to “squeeze” through gaps in the extracellular matrix. Both types of migration can be mutually interchangeable, depending on the extracellular environment (Spano et al., 2012).

Several evidence support the proposed hypothesis that VGSCs regulate the cellular movement in metastatic process; these channels modulate the cytoskeletal remodeling to facilitate the cellular movement and the extracellular matrix degradation by proteolysis, as is described in more detail in next sections. In addition, multiple quantitative measurements have revealed a depolarized membrane potential (*V*_m_) in expressing functional VGSCs cells, correlating with cellular morphology modifications and the promotion of cancer cell motility (Fraser et al., 2005; Brackenbury and Djamgoz, 2006; Brisson et al., 2013)*.* The modulation of cellular migration and directed cellular expansion by VGSCs has been previously shown mainly in developmental and neuronal processes; for example, it has been demonstrated that the neurite outgrowth promoted by the VGSCs activity is mediated by their association with the β1-subunit, Fyn kinase, and contactin (Brackenbury et al., 2008).

In PCa cells expressing functional VGSCs, treatment with TTX leads to a significant decrease in cell process length and field diameter, whereas it increases cell body diameter and process thickness, suggesting that VGSCs activity plays an important role in cytoskeleton reorganization to promote their metastatic potential (Fraser et al., 1999). In LNCaP prostate cells, a reduction in volume and a bipolar morphology are privileged when overexpressing the β2 subunit, leading to an increased migratory capability, consistent with the direct correlation between the metastatic potential and β2 expression in PCa cell lines (Jansson et al., 2012).

In MDA-MB-231 cells, the overexpression of β1 induces an increase in the process length *in vitro* (Chioni et al., 2009) and *in vivo* in a xenograft model, especially in the periphery of the tumor sections, where the cells have a more elongated morphology and are infiltrating the adjacent skeletal muscle. Like in neurons, process outgrowth mediated by the overexpression of β1 has been shown to be dependent of Fyn kinase, suggesting an augmented mesenchymal migration (Brackenbury et al., 2008; Nelson et al., 2014). In the same cells, the amoeboid cell migration is enhanced when the β4 expression is stably downregulated (Bon et al., 2016), conferring the cells the ability to squeeze and migrate through small gaps in the extracellular matrix, consistent with an augmented migration and invasiveness of these cells compared to control cells.

The expression of β4-subunit is downregulated in breast cancer, cervical cancer primary cultures and thyroid cancer (Hernandez-Plata et al., 2012; Bon et al., 2016; Gong et al., 2018), compared with non-cancerous tissues. Furthermore, in breast cancer, it has been shown that the diminished levels of β4 subunit potentiate cellular migration and invasiveness through an overactivation of the RhoA pathway, and this correlates with a high metastatic potential, as well as with a malignant phenotype. Based on these observations, the *SCN4B* gene coding for the β4 subunit has been proposed as a tumor suppressor gene, as its preserved expression reduces breast cancer tumor progression, and is considered as a clinical recurrence-free survival marker for thyroid cancer (Bon et al., 2016; Gong et al., 2018). A more recent study showed that β4 subunit may participate in the maintenance of the epithelial phenotype in mammary cells, as its downregulation promotes a complete loss of epithelial organization. This effect was associated with an increased degradation of β-catenin, reduced E-cadherin expression, and induction of mesenchymal markers such as N-cadherin, vimentin, and α-SMA. With this experimental evidence, authors conclude that β4-subunit downregulation might be a determining step in early carcinogenesis (Doray et al., 2021).

The blockade of the VGSCs activity in metastatic cancer cells causes a reduction on the cellular lateral motility; this phenomenon has typically been studied by the wound healing assay. It is the case for highly metastatic prostate cancer MAT-LyLu cells, which express functional TTX-sensitive channels (Fraser et al., 2003). Agonists of VGSCs promote the opposite effect; they increase the motility index of the cells, whereas no effect on motility is induced by the same compounds in AT-2 cells where voltage-activated sodium currents are absent (Fraser et al., 2003). Similar effects have been observed with breast cancer MDA-MB-231 cells, in the presence of TTX (Pan and Djamgoz, 2008). Primary malignant pleural mesothelioma cells migration is also reduced by incubation with TTX, analyzed by radial monolayer assays (Fulgenzi et al., 2006). Moreover, transfection with specific siRNAs targeting neonatal Na_V_1.5, which is overexpressed in astrocytoma cells, reduce the distance of migration of these cells (Xing et al., 2014), demonstrating the role of VGSCs in lateral motility. In addition, this cellular behavior is modulated by growth factors, such as EGF, NGF, insulin, and VEGF, which have regulatory effects on the VGSCs expression and activity (Pan and Djamgoz, 2008; Fraser et al., 2014b).

Not only the conducting α subunit of VGSCs can modify the lateral motility of the cancer cells, but also the VGSC β-subunits are involved in this behavior, likely related to their well-known activity as CAMs, mainly reported for prostate cancer cells. Overexpression of β2 in LNCaP cells increases cell lateral motility on plastic tissue culture dishes as well as in laminin-coated plates (Jansson et al., 2012; Jansson et al., 2014). Also, the downregulation of β4 in MDA-MB-231 cells significantly increases the lateral migration speed, evocating a transition towards the amoeboid invasiveness (Bon et al., 2016).

Another technique widely used to assess the migration capacity of cells is through transwell assays, where cell movement arises in response to chemoattractants. Typically, the transwell migration of several cancer cells expressing sodium influx carried by VGSCs is reduced in the presence of TTX or other blocking toxins. This is the case of breast (Fraser et al., 2005; Pan and Djamgoz, 2008; Chioni et al., 2009), and ovarian cancer (Gao et al., 2010). The same modulating effect can be seen using siRNAs to downregulate the expression of Na_V_1.5 in U251 astrocytoma cells (Xing et al., 2014), although in other cases, the transwell migration is not affected by the downregulation of VGSCs (Roger et al., 2007; Carrithers et al., 2009; Hernandez-Plata et al., 2012). In the same way, the response to the chemotactic signal ejected by VEGF is reduced in HUVEC cells in the presence of TTX in a dose-dependent manner (Andrikopoulos et al., 2011).

In addition, β subunits also play important roles in this type of migration. Transfection with siRNAs targeting β1 increases the *in vitro* transwell migration of breast and cervical cancer cell lines (Chioni et al., 2009; Sanchez-Sandoval and Gomora, 2019), most likely due to a reduction on the cell-cell adhesion strength, allowing the cells to move more easily. Consistent with these observations, overexpression of β1 subunit causes a significant reduction in the transwell migration of HeLa, SiHa, and CaSki cervical cancer cells (Sanchez-Sandoval and Gomora, 2019). On the other hand, the downregulation of β4 subunit increases the transendothelial migration of the breast cancer MDA-MB-231 cells, and the overexpression reverts those effects. These observations were done by testing the capacity of the cancerous cells to move across an endothelial layer and an 8-μm pore-sized filter to reach a nutrient-enriched medium. The role of β4 as a cancer suppressor protein in this cell line was associated to the overactivation of RhoA GTPase activity, and cytoskeletal reorganization promoted by the intracellular C-domain of the β4 subunit. Additionally, studies with *in vivo* models showed that the overexpression of β4 reduces metastasis of breast cancer cells to lungs (Bon et al., 2016).

Interestingly, cell movement can also be modulated by electric fields. Embryos and most organs generate transepithelial potentials due to the directional ion transportation in epithelial cells, ranging from a few to tens of millivolts. These correspond to transcellular direct-current electric fields (dcEFs) of 50–500 mV/mm, which play a significant role in major biological processes such as embryogenesis, wound healing, and tissue regeneration, directing the effective cell migration to appropriate places for proliferation and differentiation. Electric fields can be clearly identified in glandular tissues, due to the ionic gradients through the lining epithelial cells. Some cells activate motility in the presence of an electric field; this directional movement towards the cathode or the anode is called galvanotaxis, or electrotaxis. The pole to which each cellular type directs its movement is specific and is a major cellular effect of dcEFs (Nuccitelli, 1988; Mycielska and Djamgoz, 2004; Zhao, 2009; Mehta et al., 2021). Galvanotaxis modulates the cytoskeletal reorganization during cell motility via actin polymerization toward the expanding direction; also, this property modulates the formation of new adhesion points between the cell and the extracellular matrix (Yan et al., 2009).

The first demonstration about the regulation of galvanotaxis exerted by VGSCs activity was showed using the highly metastatic prostate cancer MAT-LyLu cell line. These cells strongly responded to the application of an electric field by migrating toward the cathode, whereas the weakly metastatic AT-2 cells did not respond. The galvanotactic response of the MAT-LyLu cells was suppressed by TTX treatment and enhanced by veratridine (a VGSCs activator). Both compounds had little effect on the AT-2 cells (Djamgoz et al., 2001). Similarly, galvanotactic migration (anodal in this case) of highly metastatic breast cancer MDA-MB-231 cells was greatly suppressed by TTX application and, once again, the weakly metastatic cancer cell line, MCF-7, did not show any galvanotactic migration in the presence or absence of the toxin (Fraser et al., 2005). These results are consistent with functional VGSCs expression occurring specifically in highly metastatic cells and confirm the participation of VGSCs in this behavior.

The mechanisms determining the polarity of the electromigration of cancer cells has been started to be identified. It has been shown that electromigration is controlled by activation of phosphatidyl-inositol 3 kinase (PI3K), as well as Src and JAK1 tyrosine kinases (Zhao et al., 2006). Also, it has been proposed that the electric fields activate signaling pathways on both poles of the cells, being very similar to chemotaxis in terms of signal transmission (SenGupta et al., 2021). Thus, the activation of the VGSCs on the cathodal side of the cell causes the electrical repulsion of intracellular Ca^2+^ ions by Na^+^ ions, increasing actin filament polymerization, which is regulated by gelsolin and other factors. When localized Ca^2+^ concentration decreases at the leading edge, elongation of actin filaments occurs, while in the rear end of the cell, where Ca^2+^ is increased, myosin contraction driven by ATP in the presence of calcium ions is augmented. This directed “push-pull” movement could explain galvanotactic movement of these cancer cells (Borys, 2012). Additional recent evidence linking the VGSCs activity with the cellular motility and metastatic behavior are exposed later in this review.

### 2.4 Extracellular matrix degradation

A crucial step in the progression of metastasis rest on the capacity of cancer cells to degrade the extracellular matrix, enabling the invasion through the adjacent tissue (Linder, 2007). This proteolytic activity, mediated by different enzymes, is highly dependent on the acidification of the peri-membrane extracellular environment, and is regulated by complex mechanisms in which VGSCs play important roles, which are discussed in the next 3 subsections.

#### 2.4.1 Matrix metalloproteinase activity

The matrix metalloproteinases (MMPs) are proteolytic enzymes that carry out the degradation of ECM proteins and have been found to be overexpressed and/or overactivated in almost all human cancers compared with normal tissue, regulating several cell behaviors, including cell growth, migration, invasion, immune surveillance, and angiogenesis. The MMPs are synthesized as inactive proteins (pro-MMPs) that must undergo a proteolytic cleavage mediated by other activated MMPs or serine proteinases, usually outside the cell, to originate the enzyme in the active form (Egeblad and Werb, 2002).

The VGSCs activity has been correlated with the activity of MMPs in breast and cervical cancer cells. In MDA-MB-231 cells, a 24 h treatment with TTX significantly reduced the MMP-9 mRNA levels, in a study published by Gao and colleagues (Gao et al., 2009). In another report using an orthotopic xenograft model, the number of MDA-MB-231 cells expressing MMP-9 was significantly reduced in tumors of animals treated with phenytoin, a VGSCs blocker (Nelson et al., 2015). In addition, the cervical cancer cell lines C33A, HeLa, and SiHa showed a specific increase in protein levels and activity of MMP-2 after transfection with the Na_V_1.6 channel; on the contrary, the presence of TTX in these cells prevented this increased MMP activity. Interestingly, neither MMP-9 nor cysteine cathepsins, or serin and threonine peptidases activities were affected because of the overexpression of Na_V_1.6 channel. Interestingly, this work also demonstrated an increase in expression and activity of the Na^+^/H^+^ exchanger 1 (NHE-1), which potentiates the specific activity of MMP-2 (Lopez-Charcas et al., 2018).

#### 2.4.2 pH regulation

It is well known that cancer cells have a reprogrammed energy metabolism, limiting it largely to glycolysis as the main ATP producer pathway over oxidative phosphorylation, leading to a metabolic state that has been termed ‘‘aerobic glycolysis” (Hanahan and Weinberg, 2011). A direct result of this metabolic change is the substantially increased production of lactate, which in principle should generate an intracellular acidic environment. However, cancer cells have a reversal pH gradient compared to normal tissue; an extracellular acid microenvironment linked to an alkaline intracellular pH. This pathological condition plays an important role in neoplastic progression and is driven and maintained by numerous cellular mechanisms including the upregulation of the NHE-1, which has been considered as the major factor in promoting tumor acidity (Kemp et al., 2008; Reshkin et al., 2013).

NHE-1 is upregulated in different types of cancer and plays a fundamental role in malignant invasion by altering the metabolic environment and cell invasiveness, including the activation of matrix metalloproteinases (Gillet et al., 2009; Lin et al., 2011; Lin et al., 2012; Litan and Langhans, 2015). It has been demonstrated in the highly metastatic breast cancer cell line MDA-MB-231 that Na_V_1.5 channel interacts with NHE-1 in focal extracellular matrix (ECM) degradation sites (invadopodia) *in vitro* **.** The two proteins colocalize with caveolin-1, and the Na_V_1.5 activity is responsible for the allosteric modulation of NHE-1, enhancing invadopodial proteolytic activity of cathepsins (specially cathepsin B) by the local acidification of perimembranal regions; as result, the extracellular matrix is degraded, and lipids in the plasma membrane are reorganized facilitating the cytoskeleton remodeling activating the Src kinase pathway. This ECM degradation is reduced in the presence of TTX or when the sodium channel is downregulated by siRNAs, indicating that the sodium channel activity, and not only the presence of the protein is needed to increase the NHE-1 function (Brisson et al., 2011; Brisson et al., 2013). More recently, the same research group, reported a positive relationship between SCN5A gene (encoding for Na_V_1.5 channels) overexpression in colon tumors with the cancer progression stage and poor survival prognosis for patients. In addition, the coexpression of Na_V_1.5 channels with the antiporter NHE-1 was demonstrated in colon cancer cell lines. These observations led to consider these two proteins as molecular membrane targets for treatments against the metastatic progression of colon cancer (Lopez-Charcas et al., 2022).

In 2016, Xia and colleagues reported the aberrant Na_V_1.7 expression in gastric cancer tissue samples and two gastric cancer cell lines, where the downregulation of the sodium channel by siRNAs ultimately decreased the NHE-1 expression and activity via p38 activation and the oncoprotein metastasis-associated in colon cancer-1 (MACC1) downregulation, generating an increased extracellular pH, decreased intracellular pH, reduced invasion, and less proliferation of the cells. Consistent with this, the activation of the channel by veratridine led to an increased NHE-1 expression (Xia et al., 2016).

Additionally, the increased acidity in the extracellular environment of the cancer cells can induce the apoptotic process in normal cells, leading to their death. This means that NHE-1 upregulation by VGSCs not only can increase proteolytic activity of cathepsins at the perimembrane *in vitro* but also could increase the local invasion *in vivo* by inducing apoptosis of the surrounding healthy tissue, allowing the cancer cells to encroach the space occupied by normal cells (Xu et al., 2013).

#### 2.4.3 Endocytosis

Cells use endocytosis for diverse functions, including the modulation of responses to growth factors, regulation of ion channels, receptors, and transporters expression in the plasma membrane, as well as cell invasion by a variety of pathogens. As a consequence of the genetic dysregulation generated in oncogenesis, several human cancers show abnormal expression and mutations of proteins related to endocytosis, including membranal receptors, small GTPases which control the vesicle recycling, and other components of the regulatory mechanisms (Floyd and De Camilli, 1998; Mellman and Yarden, 2013). In addition to the vast description of the endocytic process accumulated in the literature since its identification and characterization, a few years ago the modulatory role of VGSCs in this cellular process was described. Now, it is well known that the enhanced endocytic activity is related to the presence of voltage-activated sodium currents in metastatic breast, prostate, and small-cell lung cancer cell lines as revised in the following paragraphs.

The strongly metastatic prostate cancer Mat-Ly-Lu cells exhibit a higher endocytic activity than poorly metastatic AT-2 cells, and this enhanced activity seems to be dependent on the functional expression of VGSCs, as incubation with TTX induced a diminished endocytic activity in Mat-Ly-Lu cells (Mycielska et al., 2003; Krasowska et al., 2004). Interestingly, endocytic activity in human prostate cancer PC3 cells is downregulated not only by the treatment with TTX and siRNAs against VGSCs, but also by treatment with eicosapentaenoic acid (EPA, 20:5 ω-3), which promotes the downregulation of the expression of Na_V_1.6 and Na_V_1.7 (Nakajima et al., 2009). In MDA-MB-231 cells, endocytic activity is also related to VGSCs activity, measured by horseradish peroxidase uptake. This activity is reduced in the presence of TTX as well as by the removal of extracellular sodium. In the same sense, aconitine (VGSCs opener) increased endocytosis in these cells, while the weakly metastatic MCF-7 cells have a significantly weaker endocytic activity and are not affected by the TTX treatment (Fraser et al., 2005). Similar results have been reported in small-cell lung cancer cells, showing a significantly higher endocytic activity than normal airway epithelial cells, which is reduced by the treatment with the VGSCs blockers TTX, lidocaine and phenytoin, or the incubation with a sodium-free solution (Onganer and Djamgoz, 2005).

It has been proposed that the molecular mechanism regulating endocytosis by VGSCs could involve Ca^2+^ mobilization from intracellular stores (Mycielska et al., 2003). More recent evidence has shown that VGSCs activate small GTPases and trigger the cytoskeleton reorganization in local membranal regions, where they colocalize with the cytoskeletal proteins clathrine or caveolin (Brisson et al., 2012; Brisson et al., 2013). In addition, the abnormal endocytosis in cancer cells could be involved in the dissolution of cell-cell junctions and loss of morphological polarity by the internalization and unbalanced distribution of junctional proteins in the plasma membrane, contributing to cell adhesion and migration. Likewise, a growing number of evidences suggest the role of the inactivation of growth factor receptors by endocytosis in the gain of self-sufficiency in growth signals in cancer cells (Mosesson et al., 2008). There is also the possibility that endocytosis proteins could be interacting directly or indirectly with signaling proteins controlling cell proliferation and survival (Floyd and De Camilli, 1998).

### 2.5 Circulation

Entering to the bloodstream demands changes in shape and cellular volume, including variations in intracellular osmolarity. As exposed previously, VGSCs are activated with *V*_m_ depolarization, so they also participate in this cellular property, contributing to cytoskeletal reorganization, changing the ionic balance, and stablishing a *V*_m_ shifted to depolarized values compared to normal cells. Functional expression of VGSCs (both, sensitive and resistant to TTX) has been identified in leukemic cells, including cell lines (Fraser et al., 2004), and circulating cells in peripheral blood samples (Lo et al., 2012). Importantly, the blockade of sodium current decreased the invasion capacity of this cells, bolstering the relevant role of VGSCs in the founding of favorable molecular conditions to potentiate invasion (Huang et al., 2015). Also, VGSCs promote the degradation of extracellular matrix surrounding endothelial cells, facilitating the intravasation of cancer cells into the blood or lymph circulatory systems to reach distant organs and produce secondary tumors (Gillet et al., 2009; Brisson et al., 2011; Bon et al., 2016). This occurs as a response to chemotaxis exerted by growth factors, hormones, chemokines, and nutrients, as well as oxygen gradients or galvanotaxis, as has been shown in breast, cervical, lung, and prostate cancer (Djamgoz et al., 2001; Roger et al., 2003; Yan et al., 2009; Pan et al., 2012; Campbell et al., 2013; Lee et al., 2019; Alassaf and Mueller, 2020).

#### 2.5.1 Resisting apoptosis

To enter the bloodstream and to reach another tissue from the primary tumor, the cancer cells must undergo the processes of intra and extravasation and survive several stressful situations, having to withstand apoptosis to conquer distant organs. The efficiency of apoptosis induction in the early phases of intravasation constitute an important barrier to metastasis (Mehlen and Puisieux, 2006). In these characteristics, VGSCs have been shown to play important roles, as summarized below.

Tumor progression correlates with the loss of function of several pro-apoptotic signals as well as the gain of function of anti-apoptotic genes, consistent with the fact that recurrent metastatic cancers usually display increased chemoresistance, as most conventional chemotherapeutic drugs function by inducing apoptosis of cancer cells (Mehlen and Puisieux, 2006). The whole apoptotic process is normally highly controlled, and it is orchestrated by many different signals and molecules, including the VGSCs. In normal astrocytes VGSCs maintain the Na^+^/K^+^ ATPase activity, preserving the sodium, potassium, and calcium homeostasis, and their inhibition accelerates the rate to trigger apoptosis (Sontheimer et al., 1994).

In U251 astrocytoma cells, the downregulation of neonatal Na_V_1.5 expression by siRNAs increases the apoptotic rates, suggesting a role of VGSCs in preventing apoptosis (Xing et al., 2014). A similar role for Na_V_1.6 has been described in Ewing Sarcoma (ES), the second more frequent type of bone cancer. In this work, Hernandez-Muñoz and cols (Hernandez-Muñoz et al., 2016) found that ES primary tumors show high levels of RING1B, a protein that belongs to the Polycomb (PcG) family of epigenetic regulators, which catalyzes the K119 ubiquitination of histone H2A, thus resulting in gene repression (Leeb and Wutz, 2007). In ES cells, the group of Hernandez-Muñoz demonstrated that RING1B directly binds the *SCN8A* sodium channel promoter reducing Na_V_1.6 expression and function, which protects ES cells from apoptotic cell death through the downregulation of NF-κB signaling pathway. Interestingly, the depletion of RING1B in ES cells induced an enhanced Na_V_1.6 expression and function which resulted also in higher levels of apoptotic cells. The involvement of Na_V_1.6 in apoptosis-related signaling has been also reported in Follicular Thyroid Carcinoma (FTC), where the apoptosis of FTC-133 cells was enhanced upon treatment with ubenimex (an antineoplastic drug that promotes tumor cell apoptosis), and, when combined with a siRNA against Na_V_1.6, an enhanced effect was observed. These observations led the authors to propose a potential new strategy for treatment of FTC patients by downregulating Na_V_1.6 in FTC cells which may increase the sensitivity of tumor cells to antineoplastic drugs (Li et al., 2022).

Furthermore, stably β1 transfected MDA-MB-231 breast cancer cells show a significant reduction on the number of cells expressing activated caspase-3 in xenograft tumors compared to control tumors, suggesting that β1 expression enhances resistance to apoptosis (Nelson et al., 2014). However, not all VGSCs β subunits appear to have the same effect, as the exogenous overexpression of β3 in osteosarcoma Saos-2 cells and in glioblastoma T98G cells, activates the apoptotic response mediated by the tumor suppressor protein p53 (Adachi et al., 2004). Surprisingly, a recent work on hepatocellular carcinoma (HCC) found contrasting results with the pro-apoptotic function of VGSCs β3-subunit. By using HepG2, a HCC cell line, Li and cols (Li et al., 2020) demonstrated that β3 subunit knockdown induces cell cycle arrest in HepG2 cells and attenuates tumor growth in nude mice. In addition, their findings indicate that β3 could bind to p53, which promotes its ubiquitination and degradation, leading to a lower level of p53. With these observations, the authors conclude that β3 expression contributes to cell proliferation and tumorigenesis by enhancing p53 degradation thus avoiding apoptosis. These contrasting results could be due to the molecular regulation of p53 ubiquitination and degradation carried by MDM2, a negative regulator of this tumor suppressor (Koo et al., 2022). The binding of β3 to p53 could stabilize the p53/MDM2 complex promoting p53 ubiquitination and degradation (Li et al., 2020), whereas the exogenous overexpression of β3 could competitively promote the release of p53 from such complex and apoptosis in Saos-2 and T98G cells (Adachi et al., 2004). An additional study showed that β3 expression was totally absent in two metastatic BCa cell lines, whereas the other three VGSCs β-subunits were present at different levels of expression (Chioni et al., 2009). A major weak point in these three works is that the *SCN3B* levels in non-cancerous cells were never explored, which could be a useful data for discriminating among the two proposed mechanisms for the β3 role in apoptosis. In this regard, a different study reported that β3 expression was upregulated in cervical cancer biopsies compared to non-cervical cancer tissue (Hernandez-Plata et al., 2012), although the potential effects of this higher levels of β3 in the metastatic behavior of the cervical cancer cells was not investigated. Undoubtedly, additional research is required to fully clarify the mechanism associated to the role of β3 in cancer apoptosis. Regardless, these findings highlight the multi-functionality and the tissue-specific roles of VGSCs in the cancer process.

### 2.6 Invasion

The most frequently associated role of VGSCs with the metastatic cancer behavior is their participation in the enhanced capability of cancer cells to perform transversal invasion, usually analyzed *in vitro* by transwell assays, using inserts coated with a mixture of proteins simulating the extracellular matrix. In most of the cases where the expression of a specific type of VGSCs is correlated to the malignancy of cancer cells, the role of the channels is mainly throughout an increment of the invasiveness potential. This is the case for breast, cervical, colon, gastric, leukemia, melanoma, non-small cell lung, ovarian, and prostate cancer cells, where the presence of TTX reduced the number of cells capable to invade (Grimes et al., 1995; Laniado et al., 1997; Bennett et al., 2004; Fraser et al., 2004; Fraser et al., 2005; Brackenbury et al., 2007; Roger et al., 2007; Carrithers et al., 2009; Gao et al., 2009; Nakajima et al., 2009; Gao et al., 2010; House et al., 2010; Brisson et al., 2011; Hernandez-Plata et al., 2012; Brisson et al., 2013; Campbell et al., 2013; Huang et al., 2015; Xia et al., 2016; Guzel et al., 2018). In the same sense, the downregulation of VGSCs expression by siRNAs or shRNAs reduces the invasion capabilities of the cells, as shown in astrocytoma, breast, colon, leukemia, prostate, and thyroid cancer (Carrithers et al., 2009; Nakajima et al., 2009; House et al., 2010; Brisson et al., 2011; Brisson et al., 2013; Xing et al., 2014; Guzel et al., 2018; Li et al., 2022), as well as the incubation of colon cancer cells in presence of ranolazine, and endometrial cancer primary cultures with PF-05089771, another VGSCs blocker (Guzel et al., 2018; Liu et al., 2019). In the same line of research, heterologous overexpression of VGSCs in cervical, non-small cell lung, and prostate, cancer cells promotes a higher number of cells capable of invading (Bennett et al., 2004; Campbell et al., 2013; Lopez-Charcas et al., 2018), and the same behavior is observed when using the VGSCs positive modulator veratridine in colon, endometrial cancer cells and non-small cell lung cancer cell lines (Campbell et al., 2013; House et al., 2015; Liu et al., 2019).

Podosomes are membrane-associated structures specialized in the directed cellular movement promoted by actin polymerization at the leading edge of motile cells that respond to chemoattractants by secreting matrix metalloproteinases (MMPs), thus facilitating the cell detachment and cytoskeletal reconfiguration to drive cellular movement. Podosomes are generally associated with non-transformed cells involved with matrix remodeling events (i.e., osteoclasts, and vascular smooth-muscle cells), and not basement membrane invasion. In cancer cells, specialized protruded membrane regions are formed in the invasion process, and, due to the high similarity in structure and components, they have been identified as invadosomes or invadopodia. Both, podosomes and invadopodia, typically exhibit an actin bundle surrounded by several cell signaling regulators to promote motility, such as Arp 2/3, WASP, Src kinase, and cortactin among others. However, invadopodia are highly protrusive and matrix-degrading structures, specialized in directing cancer cell invasion through basement membrane (Murphy and Courtneidge, 2011; Lohmer et al., 2014; Angus and Ruben, 2019; Masi et al., 2020). The functional activity of VGSCs promotes changes in the cellular physiology, which activates cellular directional movement initiated by invadopodia in cancer cells at the beginning of the metastatic process (Brisson et al., 2012). The localized sodium influx permeated through VGSCs are coupled to the activity of other proteins colocalized in lipid rafts to potentiate the cytoskeletal reorganization, cell detach from the extracellular matrix, and invasion to surrounding tissues first and distant sites latter among them. It has been also demonstrated that NHE-1 and the Na^+^/Ca^2+^ exchanger (NCX) both have relevant functions in the invadopodia activity (Kemp et al., 2008; Khananshvili, 2013).

The molecular mechanisms underlying the participation of VGSCs in the metastatic process has not been totally clarified, but a significant amount of evidence has emerged during the last decade. The invasion capacity of cancer cells is a consequence of the simultaneous activation of several of the metastatic behaviors described above (e.g., differential migration, adhesion, activation of MMPs, etc.) together with the activation of other molecules involved in numerous cell signaling pathways and some of which have been correlated with the functional expression of VGSCs, including JAK/STAT, PKC, MAPK, NCX, PKA, and Src kinase (Chioni et al., 2010; House et al., 2010; Andrikopoulos et al., 2011; Brisson et al., 2013; House et al., 2015; Li et al., 2022).

The blockade of intracellular VGSCs in macrophages and melanoma cells prevents cellular invasion, whereas the activation of the channels by veratridine promotes it, apparently through a vesicular intracellular sodium release leading to a mitochondrial calcium release (by the NCX), activating the actin cytoskeleton dynamics, which in turn enables the rapid assembly and disassembly of podosomes and invadopodia (Carrithers et al., 2009). In MDA-MB-231 cells, the physical interaction of Na_V_1.5, NHE-1, and caveolin-1 in invadopodia has been shown by co-immunoprecipitation assays. The activation of Na_V_1.5 increases Src kinase activity, modifies F-actin polymerization and thus the regulation of the invadopodia formation (Brisson et al., 2013).

Among the hypothesis proposed to explain the molecular mechanisms by which VGSCs promote metastasis, one of them proposes the activation of cytoskeleton reorganization triggering the actin polymerization via PKA activity. Nevertheless, the precise connection between VGSCs activity and phosphorylation cascades activation was missing until a few years ago. Recent evidence has been added to clarify the molecular mechanism. The functional expression of any VGSC type in cancer cells stablish a more depolarized *V*_m_ in comparison with no cancerous cells from the same tissue; furthermore, it has been shown that the most depolarized cells in breast cancer have the highest metastatic potential (Fraser et al., 2005; Yang and Brackenbury, 2013). In the highly metastatic breast cancer cells MDA-MB-231, the membrane depolarization stablished by the Na_V_1.5 activity induces a phospholipid redistribution, including phosphatidyl inositol bisphosphate (PIP2); and phosphatidylserine, which interacts with the small GTPase Rac1 (anchored to the plasma membrane) to trigger the activation of Rac1 and Src kinases which in turn prompts actin filament polymerization and cytoskeletal reorganization through phosphorylation and positive modulation of the Arp2/3 complex, cortactin and cofilin at the leading edge of the cell; thus boosting the plasma membrane protrusions, which promotes the acquisition of a cell motile and mesenchymal-like cellular phenotype (Brisson et al., 2013; Augoff et al., 2020; Yang et al., 2020). The activity of small GTPases Rac1, Rho, and cdc42 reconfigures the cytoskeleton organization promoting the directed cellular movement and participating in the assembly of specialized motility structures in the cell membrane, the lamellipodia and invadopodia (Burridge and Wennerberg, 2004; Wu et al., 2009). In the same way, the small GTPase K-Ras is also activated because of *V*_m_ depolarization and the subsequent redistribution of phosphatidylserine (Zhou et al., 2015), triggering the cell membrane reorganization and other cellular responses.

The role of β subunits of VGSCs in this behavior is less understood. β1 and β2 expression correlates with the invasive potential in prostate cancer cells. The overexpression of β2 in LNCaP cells promotes a 3.5-fold increase of transwell invasion (Diss et al., 2008; Jansson et al., 2012). On the contrary, in breast cancer cells, β1 expression is inversely correlated with the native invasiveness potential of the cells (Chioni et al., 2009), although, overexpressing this subunit in MDA-MB-231 cells enhances the *in vitro* transwell invasion, as well as the *in vivo* tumor growth, suggesting the role of β1 as CAM via a *trans*-homophilic adhesion mechanism that improves process outgrowth as described before, enabling the cell dissemination into surrounding tissues (Nelson et al., 2014). In the same sense, downregulation of β1 or β2 expression by siRNAs decreases invasiveness in these cells, while the treatment with siRNAs against β4 have the opposite effect, as it also does in cervical cancer cell lines, in accordance with the lower or lack of expression of β4 in breast and cervical cancer biopsies compared with normal tissue (Bon et al., 2016; Sanchez-Sandoval and Gomora, 2019). Loss of β4 expression in cancer cells seems to stimulate their invasiveness through the enhanced activation of the RhoA pathway, favoring amoeboid migration of the cells, and the amplitude of *I*_Na_P (Bon et al., 2016).

## 3 Molecular mechanisms underlying metastasis promoted by VGSCs

**FIGURE 3:**
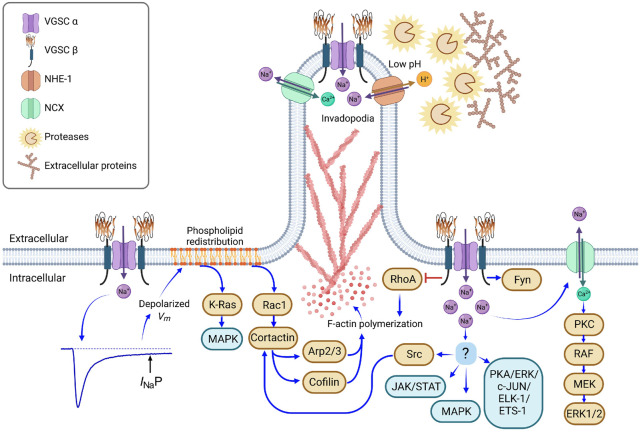
Molecular mechanisms involved in the metastatic behavior promoted by VGSC. Hypoxic conditions in growing tumors promote the incomplete inactivation of VGSC, generating a persistent sodium current (*I*_*Na*_P), which induces a depolarized resting membrane potential (*V*_*m*_), as has been described with Na_V_1.5 activity in breast and colon cancer cells (Djamgoz and Onkal, 2013; Guzel et al., 2018). Depolarized *V*_*m*_ stimulates the phospholipid redistribution; reorganization of phosphatidylinositol-bisphosphate and phosphatidylserine activates Rac1 and K-Ras small GTPases, fomenting the F-actin polymerization through the activation of cortactin, Arp2/3 and cofilin, and the activation of the MAPK signaling pathway (Zhou et al., 2015; Yang et al., 2020). On the other hand, VGSC α subunits interact physically with β subunits, NHE-1, and NCX antiporters in localized membranal regions. VGSC activity promotes the activation of NHE-1 and, as a result, the extracellular perimembrane pH is acidified, favoring the protease activity (cathepsins and matrix metalloproteinases) and the degradation of anchoring-extracellular proteins at the leading edge of motile cells (Brisson et al., 2013; Lopez-Charcas et al., 2018). VGSCs also promote the Src kinase activity and the subsequent F-actin polymerization through the phosphorylation of cortactin (Brisson et al., 2013), as well as the activation of the MAPK, JAK/STAT and PKA signaling pathways, involved in cell proliferation, epithelial-mesenchymal transition and invasion (Chioni et al., 2010; House et al., 2015; Li et al., 2022). Increments in local sodium concentration drive to the reversed function of NCX, pumping sodium out and transporting calcium (Ca^2+^) into the cell. In turn, Ca^2+^ inside the cell activates PKC kinase, following by the activation of RAF, MEK and ERK1/2, which are involved in cell proliferation, as reported in endothelial cells (Andrikopoulos et al., 2011). VGSC β subunits also interact with a variety of molecules. β1 has been shown to promote the process outgrowth in breast cancer cells through the activation of a signaling cascade via Fyn kinase (Brackenbury et al., 2008; Nelson et al., 2014), whereas the activity of RhoA in F-actin polymerization is inhibited by the physical interaction with β4 (Bon et al., 2016). Brown boxes indicate a specific molecule, blue boxes stand for signaling pathways. Figure created using BioRender.

The mobilization of cancer cells during the invasion and metastatic process to surrounding and distant sites demands changes in the gene expression profile, being one of the most typical changes the epithelial-to-mesenchymal transition (Pastushenko et al., 2018). As part of the process, the cell displays polarization and invadopodia formation with evident membranal protrusions at the leading edge, where normally the cell responds to chemoattractants (Figure 3). Additionally, cancer cells also modify their gene expression profile in response to low oxygen concentrations, which normally increases the metastatic potential (Sforna et al., 2014; Li et al., 2015; Chen et al., 2021; Novin et al., 2021).

The functional expression of VGSCs in the modulation of cell migration has been revealed in cancerous cells from different tissues (Black and Waxman, 2013). In breast cancer cells, Na_V_1.5 channels are upregulated; in addition to migration, they promote invasion and metastasis (Roger et al., 2003; Fraser et al., 2005; Yildirim et al., 2012; Nelson et al., 2015). The molecular mechanism by which these channels exert the effect remains as one of the priority goals in the field. The coexpression of VGSCs and other molecular components of the invadopodia machinery in protrusions of the plasma membrane in migrating cells has been proved (Brisson et al., 2011; Besson et al., 2015). More recently, in breast cancer cells it has been described a molecular mechanism involving a signaling pathway where sodium influx throughout the Na_V_1.5 channels activates the cytoskeleton reorganization and promote cellular mobilization, as it happens in the metastatic process. The MDA-MB-231 human breast cancer cells express a persistent sodium current which leads to the reorientation of phosphatidylserine and activates the small GTPase Rac1. In turn it triggers a cascade of phosphorylations leading to the activation of Arp2/3, cortactin, and cofilin, promoting the motile phenotype of the cells (Yang et al., 2020)*.* Rac1 is intracellularly anchored to the plasma membrane through a prenylation in the C-terminus; it interacts with the phospholipids PIP2, PIP3, and phosphatidylserine to be activated (Finkielstein et al., 2006; Remorino et al., 2017). Depolarization of *V*_m_ also promotes the small GTPase K-Ras activation, in response to reorganization of membrane phospholipids (Zhou et al., 2015).

The crucial participation of Na_V_1.5 channels in the modulation of *V*_m_ has been proved by blocking their expression with siRNAs (Nelson et al., 2015; Yang et al., 2020). When VGSCs are expressed in cancer cells, the *V*_m_ is more depolarized than non-expressing VGSCs cells; this generates a “responsive state” in cancer cells characterized by high metastatic potential and quick responses to chemoattractant signals. Importantly, the blockade of Na_V_1.5 channels expression with a specific siRNA diminishes the metastatic potential of breast cancer cells to lungs; the same effect was observed applying the drug Ranolazine, which blocks specifically Na_V_1.5 channels (Driffort et al., 2014)**.** Ranolazine inhibits the late sodium current and produces a reduction in the intracellular calcium concentration in cardiomyocytes; this drug is used to treat effectively cardiac dysfunctions such as angina and coronary microvascular dysfunction (Aldiwani et al., 2021; Sharp et al., 2021). The blockade of Na_V_1.5 channels suppresses Rac1 activation, reducing the cytoskeletal reorganization and cellular migration, showing a link between electrical changes in the plasma membrane and the cytoskeletal reorganization, connecting with the acquisition of the mesenchymal cellular phenotype and metastatic behavior (Yang et al., 2020).

Most recent evidence demonstrates the relevance of the elevated Na^+^ concentration in BCa tumors for predicting malignancy. By using magnetic resonance imaging (MRI) of non-invasive sodium [^23^Na], the group led by Dr. Brackenbury at the University of York showed that sodium concentration [Na^+^] in tumors was more elevated than non-tumor regions. Interestingly, they found in *ex-vivo* isolated tumor slices that the [Na^+^] was altered only at the intracellular side whereas the extracellular [Na^+^] remain unchanged. Interestingly, specific clinical-approved inhibitors of Na^+^ influx (cariporide for NHE-1; and eslicarbazepine acetate for VGSCs) did not show effects on tumor [Na^+^], but the chemotherapeutic drug docetaxel did (James et al., 2022). These results suggest that alternative Na^+^-dependent transporters might be responsible for elevated tumor [Na^+^], although authors did not rule out the possibility of fine, localized changes in Na^+^ away from the sensitivity of their MRI approach. Further work is required to establish whether these observations in BCa tumors related to the carriers responsible for the changes in Na^+^ concentration are conserved in tumors derived from different type of cancers.

## 4 Clinical potential of VGSCs in diagnosis, prognosis and treatment of cancer

The increasing evidence about the differential expression of several VGSCs α and β subunits in cancer cells compared with normal tissue rises the possibility of using these molecules as molecular markers in the detection of malignant cells, as well as molecular targets for a more accurate treatment. As it has been mentioned before, neonatal and other splice variants of the channels are preferentially expressed in cancer, and this fact leads to the possibility of designing strategies (e.g., antibodies or drugs) directed towards these isoforms, without altering the functionality of “normal” isoforms expressed in healthy tissues, as it has been proposed for breast, cervical, colon, and gastric cancer among others (Fraser et al., 2005; Xia et al., 2016; Guzel et al., 2018; Lopez-Charcas et al., 2018).

It has been suggested that the galvanotactic and electrophysiological characteristics could be useful for diagnosis, as the electrical properties of tissues change in malignancy. Likewise, electrotherapy for some strongly galvanotactic carcinomas could work by applying small direct-current voltages in order to draw the cancer cells out, or by reducing the endogenous transepithelial potentials that facilitate galvanotaxis *in vivo*, using cellular ion pumps and exchangers inhibitors (Mycielska and Djamgoz, 2004).

Therapeutic effect of dietary components including polyunsaturated omega-3 fatty acids like docosahexaenoic acid (DHA, 22:6 ω-3) and eicosapentaenoic acid (EPA, 20:5 ω-3) might be linked to their effects on VGSCs activity. DHA blocks VGSCs activity and expression, and thus several metastatic behaviors of MDA-MB-231 cells *in vitro* using dietary-ranged concentrations of the fatty acid (Isbilen et al., 2006; Wannous et al., 2015). Something similar was reported with EPA in rat and human prostate cancer cell lines (Nakajima et al., 2009), which could explain at least some of the anticancer effects of the polyunsaturated fatty acids.

Another dietary component, the resveratrol, a natural phenolic compound found in red grapes, has been proposed as a natural antimetastatic agent, as it inhibits metastatic cell behaviors *in vitro* by blocking VGSCs activity, demonstrated in rat prostate Mat-LyLu cells (Fraser et al., 2014c).

The role of celecoxib in chemoprevention has been proposed for colorectal cancer, via induction of intrinsic pathway of apoptosis through NHE-1 activity and intracellular calcium homeostasis (Chun and Surh, 2004; Vaish and Sanyal, 2012), which might be mediated by VGSCs inhibition, as it has been demonstrated that celecoxib suppresses Na_V_1.5 currents in a dose-dependent manner (Frolov et al., 2011). This evidence has a physiological relevance as Na_V_1.5 has been reported as the VGSC involved in promoting the colorectal cancer invasiveness (House et al., 2010; House et al., 2015). In the same way, other drugs and nutritional components taken from diet have been identified as VGSCs blockers, and their utility in the treatment of cancer has been revealed recently (Lopez-Charcas et al., 2021).

On the other hand, several retrospective studies have suggested that the application of local anesthetics (specifically sodium channel blockers, like lidocaine) during cancer surgeries can reduce the chance of subsequent tumor recurrence (Tavare et al., 2012; Mao et al., 2013; Niwa et al., 2013; Eren et al., 2015). It has been proposed the use of this local anesthetics during and post-surgery to reduce the ability of the cancer cells to metastasize by inhibiting the VGSCs activity, with the advantage that these drugs are already approved for clinical use (Djamgoz and Onkal, 2013; Fraser et al., 2014a).

Surgical protocols involving general anesthesia and opioids produce perioperative immunosuppression and could result in cancer recurrence and diminish survival (Kim et al., 2022). Opioids promote tumor recurrence cancer cell proliferation and metastasis by means of their angiogenic stimulating effect on endothelial cells, inducing neovascularization (Wang et al., 2021). It has been shown that silencing the expression of opioid receptors reduces the malignancy of lung cancer cells, and the opposite effect is generated when these receptors are overexpressed (Mathew et al., 2011; Lennon et al., 2014).

In addition, non-opioids anesthetics, such as lidocaine can induce apoptosis in breast cancer cells, highlighting its utility as metastatic suppressors in surgical procedures (Chang et al., 2014)**.** Indeed, some studies have identified that the blockade of VGSCs with anesthetics, antiepileptics or other drugs, improves the evolution of patients after anti-oncological treatment (Fairhurst et al., 2015; Martin et al., 2015).

Cancer cells express mainly neonatal variants of VGSCs. Local anesthetics, lidocaine and phenytoin induce a greater blockade of the neonatal Na_V_1.5 and Na_V_1.7 isoforms. In addition to the invasion and metastatic potential inhibition, local anesthetics diminish proliferation and tumor growth (Fedder et al., 2010; Lucchinetti et al., 2012). The anesthetics lidocaine, bupivacaine, and ropivacaine, used in surgical procedures, inhibit VGSCs and affect proliferation (Fedder et al., 2010; Baptista-Hon et al., 2014). Also, it has been recently shown the molecular mechanism by which the anticonvulsant lacosamide inhibits the Na_V_1.7 channels (Labau et al., 2021); so, it would be interesting to evaluate its effect on prostate cancer, where the overexpression of Na_V_1.7 channels is directly related to the tumor expansion and aggressiveness (Diss et al., 2001; Yildirim et al., 2012).

Concerning the molecular mechanisms, some local anesthetics, such as lidocaine and phenytoin, inhibit the endocytic activity promoted by VGSCs in lung cancer cells with a concentration-dependent correlation (Onganer and Djamgoz, 2005). Also, it has been shown that local anesthetics inhibit the Src signaling pathway, which results in a diminished motility and metastatic behavior (House et al., 2010; Piegeler et al., 2012). In ovarian cancer, lidocaine inhibits the cellular malignant potential by blocking the phosphorylation-dependent activation of FAK/paxillin signaling pathway (Liu et al., 2020). In colon cancer, propofol decreases cell invasiveness, reducing the extracellular matrix protein expression, via the MAPK signaling pathway, involving JNK, p38, and the phosphorylation of ERK1/2 (Miao et al., 2010). In breast cancer cells it has been shown that lidocaine and tetracaine inhibit the correct assembly of the motility machinery disrupting the alpha tubulin and vimentin filaments, resulting in the inhibition of migration and invasiveness (Yoon et al., 2011). Relevantly, local anesthetics also affect other ion channels. As a consequence, their effect also includes the inhibition of proliferation, likely throughout their effect on calcium, potassium or other cationic channels (Grandhi and Perona, 2020); as well as cytokine receptors (Sakaguchi et al., 2006).

Epithelial ovarian cancer cells also express VGSCs. A systematic review of expression microarrays and RNAseq analysis showed that ovarian cancer cell lines express lower levels of VGSCs than epithelial cells from fallopian tube or control skin fibroblasts. Interestingly, a low mRNA expression of *SCN8A* (coding for Na_V_1.6 channels) was associated with a longer survival in patients with ovarian tumors, whereas a low expression of *SCN1B* (coding for the Na_V_β1 subunit) was associated with a lower survival in the same patients (Brummelhuis et al., 2021), similar to what has been reported in breast cancer for *SCN4B* (Bon et al., 2016). The quantification of VGSCs expression levels in cancer cells could be used as a prognostic factor, based on the fact that low mRNA levels of α subunits are characteristically related to high survival expectancies (except for the case of Ewing sarcoma; Hernandez-Muñoz et al., 2016); while low levels of β subunits are indicative of malignancy and highly probable metastatic cells (Fairhurst et al., 2015; Bon et al., 2016; Brummelhuis et al., 2021).

Lidocaine, and other local anesthetics increase the sensitivity to chemotherapy (carboplatin and paclitaxel) in ovarian cancer cells (Altamura et al., 2021; Brummelhuis et al., 2021). In the same way anti-epileptic drugs have shown beneficial effects employed as anti-cancer therapy, reflecting the relevance of their inclusion in antitumoral treatments (Yang et al., 2012; Fairhurst et al., 2015; Lee et al., 2019).

Interestingly, Na_V_1.6 suppressed the antineoplastic sensitivity of FTC cells *in vitro* to ubenimex, a clinically used immunopotentiator for patients, which lead authors to propose Na_V_1.6 channel as a potential target for FTC therapy (Li et al., 2022).

Recently, it has been noted that the neonatal Na_V_1.5 channels can be selectively blocked by a specific antibody, and two arachnid toxins, with little or no effect on the adult isoform (Fraser et al., 2022). These observations highlight the advance in the identification of crucial elements for the development of new therapeutical agents to treat oncological pathologies targeting VGSCs.

## 5 Concluding remarks

The functional expression of VGSCs promotes a depolarized steady state which triggers a localized activation of Rac1 that drives cytoskeletal reorganization, the generation of membrane protrusions and directed cellular movement, coupling the changes in electric fields generated by ion flow through ion channels with the modification of the cytoskeleton at the leading edge of cell.

The evidence collected so far indicate that the functional expression of VGSCs modifies the *V*_m_ in cancer cells driving them to a more depolarized state in which these cells classically acquire an elongated morphology, with membrane protrusions typically observed in invadopodia and an exacerbated activity of extracellular-matrix proteases that stimulate the migration and invasive potential.

Surgical manipulation of tumors adds on a risk of spreading malignant cells into the circulation, thus increasing the chance of secondary tumor formation. The use of local non-opioid anesthetics could avoid this phenomenon by blocking VGSCs and other ion channels, disabling tumor cells by diminishing their survival, invasiveness, and resistance to chemotherapy capacities. So, to abolish the metastatic risk because of a surgery, it is more beneficial to use non-opioid drugs which, in addition to reduce the invasive potential of cancer cells by affecting the VGSCs functionality, they also act on other ion channels diminishing proliferation and other properties of cancerous cells. Also, the quantification of VGSCs expression levels in cancer cells could be used as a prognostic factor of survival in patients with oncologic ailments; and studies focused on the identification of specific properties of VGSCs variants expressed in cancer will lead to the development of better drugs to treat cancer.

The last three decades have been of a remarkable breakthrough for the study of VGSCs’ contribution to the carcinogenesis, and significant breakthroughs in the associate mechanisms have been achieved. Nevertheless, still more work is needed to fully understand the whole signaling machinery that underlie the role of both, α and β subunits, in these new non-canonical functions that locate the VGSCs in the spotlight for cancer diagnostics and therapeutics.
